# Prophylactic Oral Dextrose Gel for Newborn Babies at Risk of Neonatal Hypoglycaemia: A Randomised Controlled Dose-Finding Trial (the Pre-hPOD Study)

**DOI:** 10.1371/journal.pmed.1002155

**Published:** 2016-10-25

**Authors:** Joanne Elizabeth Hegarty, Jane Elizabeth Harding, Gregory David Gamble, Caroline Anne Crowther, Richard Edlin, Jane Marie Alsweiler

**Affiliations:** 1 Liggins Institute, University of Auckland, Auckland, New Zealand; 2 Newborn Services, National Women’s Health, Auckland, New Zealand; 3 School of Population Health, University of Auckland, Auckland, New Zealand; 4 Department of Paediatrics: Child and Youth Health, University of Auckland, Auckland, New Zealand; University of Manchester, UNITED KINGDOM

## Abstract

**Background:**

Neonatal hypoglycaemia is common, affecting up to 15% of newborns, and can cause brain damage. Currently, there are no strategies, beyond early feeding, to prevent neonatal hypoglycaemia. Our aim was to determine a dose of 40% oral dextrose gel that will prevent neonatal hypoglycaemia in newborn babies at risk.

**Methods and Findings:**

We conducted a randomised, double-blind, placebo-controlled dose-finding trial of buccal dextrose gel to prevent neonatal hypoglycaemia at two hospitals in New Zealand. Babies at risk of hypoglycaemia (infant of a mother with diabetes, late preterm delivery, small or large birthweight, or other risk factors) but without indication for admission to a neonatal intensive care unit (NICU) were randomly allocated either to one of four treatment groups: 40% dextrose at one of two doses (0.5 ml/kg = 200 mg/kg, or 1 ml/kg = 400 mg/kg), either once at 1 h of age or followed by three additional doses of dextrose (0.5 ml/kg before feeds in the first 12 h); or to one of four corresponding placebo groups. Treatments were administered by massaging gel into the buccal mucosa. The primary outcome was hypoglycaemia (<2.6 mM) in the first 48 h. Secondary outcomes included admission to a NICU, admission for hypoglycaemia, and breastfeeding at discharge and at 6 wk. Prespecified potential dose limitations were tolerance of gel, time taken to administer, messiness, and acceptability to parents. From August 2013 to November 2014, 416 babies were randomised. Compared to babies randomised to placebo, the risk of hypoglycaemia was lowest in babies randomised to a single dose of 200 mg/kg dextrose gel (relative risk [RR] 0.68; 95% confidence interval [CI] 0.47–0.99, *p* = 0.04) but was not significantly different between dose groups (*p* = 0.21). Compared to multiple doses, single doses of gel were better tolerated, quicker to administer, and less messy, but these limitations were not different between dextrose and placebo gel groups. Babies who received any dose of dextrose gel were less likely to develop hypoglycaemia than those who received placebo (RR 0.79; 95% CI 0.64–0.98, *p* = 0.03; number needed to treat = 10, 95% CI 5–115). Rates of NICU admission were similar (RR 0.64; 95% CI 0.33–1.25, *p* = 0.19), but admission for hypoglycaemia was less common in babies randomised to dextrose gel (RR 0.46; 95% CI 0.21–1.01, *p* = 0.05). Rates of breastfeeding were similar in both groups. Adverse effects were uncommon and not different between groups. A limitation of this study was that most of the babies in the trial were infants of mothers with diabetes (73%), which may reduce the applicability of the results to babies from other risk groups.

**Conclusions:**

The incidence of neonatal hypoglycaemia can be reduced with a single dose of buccal 40% dextrose gel 200 mg/kg. A large randomised trial (Hypoglycaemia Prevention with Oral Dextrose [hPOD]) is under way to determine the effects on NICU admission and later outcomes.

**Trial Registration:**

Australian New Zealand Clinical Trials Registry ACTRN12613000322730

## Abstract 2

Jane Marie Alsweiler and colleagues report on their trial on administering oral dextrose in newborns and the management of hypoglycaemia, which is a common and potentially dangerous condition.

## Author Summary

**Why Was This Study Done?:**

- Neonatal hypoglycaemia is common, with 30% of babies born at risk for hypoglycaemia and half of these developing low blood-glucose concentrations.
- Babies who develop hypoglycaemia are at increased risk of neurodevelopmental impairment.
- There are no strategies, other than feeding the babies formula, for preventing hypoglycaemia in at-risk babies,
- Oral dextrose gel has been shown to be effective at treating neonatal hypoglycaemia.

**What Did the Researchers Do and Find?:**

- The authors conducted a randomised controlled trial of prophylactic oral dextrose gel in babies at risk of developing neonatal hypoglycaemia (infants of mothers with diabetes, small, large, and preterm) to determine the most effective dose of oral dextrose gel to reduce the incidence of hypoglycaemia.
- Four hundred and sixteen at-risk babies were randomised to receive either a standard (200 mg/kg) or high (400 mg/kg) dose of dextrose gel or placebo, either once or followed by three more standard doses before feeds.
- The authors found that 200 mg/kg (0.5 ml/kg of 40% dextrose gel) was effective at reducing the incidence of hypoglycaemia (relative risk [RR] 0.68; 95% confidence interval [CI] 0.47–0.99, *p* = 0.04).

**What Do These Findings Mean?:**

- A single dose of prophylactic oral dextrose gel reduces the incidence of neonatal hypoglycaemia in babies born at risk.
- Further research is needed to determine if oral dextrose gel also reduces rates of neonatal intensive care unit (NICU) admission and neurodevelopmental impairment.
- Most of the babies in the trial were babies of mothers with diabetes, so these results may not be as applicable to babies from other risk groups.

## Introduction

Approximately 30% of newborn babies require multiple blood tests for screening for neonatal hypoglycaemia under current guidelines. Half of these will develop hypoglycaemia [1], and an unknown proportion will experience brain damage and developmental delay as a result. Despite recommendations in clinical guidelines that prophylactic measures should be taken in babies at risk of neonatal hypoglycaemia [2–4], there currently are no strategies beyond early feeding for prevention [5]. Dextrose gel has been shown to be effective in treating neonatal hypoglycaemia, without detrimental effect on breastfeeding [6]. We therefore considered that it might also be effective as prophylaxis against neonatal hypoglycaemia. However, we first needed to determine an effective dose of dextrose gel to prevent neonatal hypoglycaemia.

A dose of 200 mg/kg glucose is the standard treatment dose of intravenous glucose administered as a “mini bolus” to babies with hypoglycaemia [7] and is also the dose demonstrated to be effective in treatment of neonatal hypoglycaemia with dextrose gel [6]. However, we considered that a single dose of 200 mg/kg glucose might not be adequate for prevention of hypoglycaemia, as babies at risk may have a prolonged nadir in blood glucose after birth [8–10] and higher plasma insulin concentrations and lower rates of hepatic glucose production in the first hours after birth than those not at risk [8]. We therefore also investigated both a higher single dose (400 mg/kg) and repeated doses in the first 12 h. Babies were randomised to the resulting eight dosage groups. The primary outcome was neonatal hypoglycaemia in the first 48 h.

### Aim

The aim of this study was to determine a dose of 40% oral dextrose gel that will prevent neonatal hypoglycaemia in newborn babies at risk.

## Methods

The trial was approved by the Northern A Health and Disability Ethics Committee of New Zealand (13/NTA/8).

### Trial Design

We undertook this randomised, double-blind, placebo-controlled trial at two hospitals providing maternity and neonatal services (Auckland City Hospital and Waitakere Hospital) in Auckland, New Zealand. Eligible babies were infants of mothers with diabetes (any type of diabetes), late preterm (35 or 36 wk gestation), small (birthweight < 10th centile on population or customised birthweight charts or < 2.5 kg) or large (birthweight > 90th centile on population or customised birthweight charts or > 4.5 kg), or those with other risk factors (e.g., maternal medication such as β-blockers). Babies also satisfied all of the following inclusion criteria at the time of randomisation; ≥35 wk gestation, birthweight ≥ 2.2 kg, < 1 h old, no apparent indication for admission to a neonatal intensive care unit (NICU) (this included a special care baby unit), unlikely to require admission to a NICU for any other reasons, and mother intending to breastfeed. Exclusion criteria were major congenital abnormality, previous formula feed or intravenous fluids given, previous diagnosis of hypoglycaemia, admitted to a NICU, or imminent admission to a NICU. Mothers of babies who were likely to become eligible (maternal diabetes, likely late preterm birth, or anticipated high or low birth weight) were identified through lead maternity carers and antenatal clinics and provided with an information sheet before the birth. Written informed consent was obtained before the birth by a member of the research team and confirmed verbally after the birth.

The trial was prospectively registered with the Australian New Zealand Clinical Trials Registry, number ACTRN12613000322730. The study protocol is available online at https://researchspace.auckland.ac.nz/handle/2292/25006.

### Randomisation

**Fig 1:**
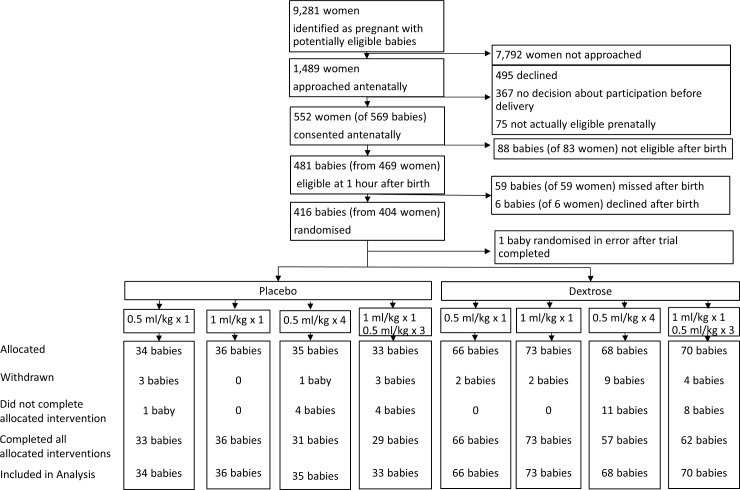
Consolidated Standards of Reporting Trials (CONSORT) diagram. One baby was randomised in error following closure of the trial after randomisation of 415 babies and was excluded from the analysis. All other babies had primary outcome data available and were included in the intention-to-treat analysis.

Eligible babies for whom consent had been obtained were randomised within the first hour after birth. We used computer-generated blocked randomisation with variable block sizes to assign babies to one of eight treatment arms. Allocation was to either 40% dextrose or placebo gel and to one of the following dose regimens: 0.5 ml/kg (200 mg/kg) once, 1 ml/kg (400 mg/kg) once, 0.5 ml/kg for four doses (total 800 mg/kg), 1 ml/kg once followed by 0.5 ml/kg for a further three doses (total 1,000 mg/kg) (Fig 1). The allocation ratios were dextrose:placebo 2:1, with the intention that the placebo arms would be combined for analysis, single:multiple dose 1:1, and low:high dose 1:1. Randomisation was stratified by centre and then by prioritised allocation to primary risk factor—i.e., if more than one risk factor was present, primary risk factor was allocated in the order of maternal diabetes, preterm, small, large, or other. For example, a baby who was both preterm and whose mother had diabetes was allocated the primary risk factor of maternal diabetes. We assigned twins independently. Research staff entered demographic and entry criteria data into an online randomisation website that provided a number corresponding to a numbered trial pack that contained either a single 5 ml prefilled syringe of either 40% dextrose gel or an identical-appearing placebo (2% hydroxymethylcellulose) or four numbered syringes of gel (1 x 5 ml and 3 x 2.5 ml, all containing either dextrose or placebo gel). Clinicians, families, and all study investigators were masked to treatment group allocation throughout the study and remain so for the planned follow-up.

### Interventions

Study gel was massaged into the buccal mucosa, either once at 1 h of age (0.5 ml/kg or 1 ml/kg) or an additional three times (0.5 ml/kg) before feeds in the first 12 h, with gel given no more frequently than every 3 h. Each dose of gel was followed by a breastfeed. Feeding was according to standard hospital guidelines, which included breastfeeding within 1 h after birth and then on demand not less than every 3 to 4 h. Supplemental formula was not given routinely but could be given on parent or clinician decision.

We measured blood glucose concentrations first at 2 h after birth. Subsequent blood glucose measurement was according to the local hospital protocol, with prefeed blood glucose measurements every 2 to 4 h for at least the first 12 h, and until there were three consecutive blood glucose concentrations of ≥2.6 mM. Babies who developed hypoglycaemia were managed by the hospital clinical team according to the standard clinical practice at each site, including supplementation with formula, treatment with 40% dextrose gel, and admission to a NICU for intravenous dextrose if required.

All blood glucose concentrations were measured in whole blood by the glucose oxidase method, either with a portable blood glucose analyser (i-STAT, Abbott Laboratories, Abbott Park, Illinois, United States) or a combined metabolite/blood gas analyser (e.g., ABL 700, Radiometer, Copenhagen, Denmark).

Babies whose parent(s) gave consent had a continuous glucose monitor sensor (iPRO2, Medtronic, MiniMed, Northridge, California, US) inserted subcutaneously into the lateral aspect of the thigh as soon as possible after birth. Interstitial glucose concentrations cannot be viewed on this monitor in real time and therefore were not available to clinicians and had no influence upon clinical management. The sensors remained in situ for at least 48 h. These data (*n* = 137) will be reported separately.

Parents were contacted at 3 d and 6 wk after birth to determine feeding method and were asked about any health events since discharge and parental perceived treatment allocation on the second occasion.

### Outcomes

The primary outcome was hypoglycaemia, defined as any blood glucose concentration < 2.6 mM in the first 48 h after birth. Secondary outcomes were admission to a NICU (defined as admission for > 4 h); admission to a NICU for hypoglycaemia; hyperglycaemia (blood glucose concentration > 10 mM); breastfeeding at discharge from hospital (full or exclusive); received any formula prior to discharge from hospital; formula feeding at 6 wk of age; cost of care until discharge home (to be reported separately), and maternal satisfaction at 6 wk. An independent data monitoring committee undertook interim analyses after 120, 240, and 360 babies had been randomised and recommended that recruitment continue. The safety monitoring committee received reports of serious adverse events (seizures and death) and other adverse events: hyperglycaemia, late hypoglycaemia (blood glucose concentration < 2.6 mM for the first time after 12 h of age), delayed feeding (failure to establish breastfeeding without supplements by the end of day 3), and systemic sepsis [9].

Limitations of the trial intervention were defined as tolerance of gel (small spill [few drops], moderate spill [half of volume administered], and large spill [all of volume administered]) assessed by the clinician at time of administration, length of time to administer dose (time to massage gel into baby’s buccal mucosa), messiness (parental report), hyperglycaemia, late hypoglycaemia, delayed feeding, and acceptability of trial intervention to parent(s) (acceptable, some inconvenience, major inconvenience, or unacceptable).

### Statistical Analysis

#### Power and sample size

Based on our previous studies [1], we estimated that 50% of eligible babies would experience hypoglycaemia. We designed the trial to have 80% power to detect a 25% absolute reduction (relative reduction of 50%) in the incidence of neonatal hypoglycaemia from 50% to 25% (two-sided, alpha = 0.05). Four placebo groups were required to mask the difference in gel volume between the lower and higher initial doses of gel. However, we anticipated combining the two single dose placebo groups for analysis and doing the same for the two multiple dose placebo groups. Therefore, we required half the number of babies in each of the four placebo arms and allowed for a 5% dropout rate, giving a total sample size of 415 babies (66 in each treatment arm, 33 in each placebo arm).

#### Selection of the optimal dose

We anticipated that the selection of the optimal dose for prevention of hypoglycaemia would be based on a combination of the dose required for adequate efficacy with a minimal burden of side effects (expected to be uncommon), together with pragmatic consideration of the ease of administration, simplicity, quantitative evaluation of weighted tolerance of the intervention scores (limitations), and cost.

To visualise the association between dose and outcome, the cumulative administered dose for the single and multiple dose arms (i.e., 0, 200, 400, 800, and 1,000 mg/kg) was plotted as the independent variable against reduction in the proportion of babies with at least one episode of hypoglycaemia, summarised as a percentage (± 95% confidence interval [CI], binomial method), and plotted as the dependent variable.

The response to single placebo and multiple placebo doses was compared, and the placebo arms were pooled into a single “0” mg/kg dose. Logistic regression modelling was then used to estimate the odds of hypoglycaemia for each cumulative dose of glucose, adjusted for risk factors for hypoglycaemia (i.e., sex, gestational age, and mode of delivery). Inspection of this function was used to determine which dose(s) at the upper limit of the 95% CI excluded a 50% reduction (the prespecified efficacy endpoint), i.e., a difference in odds of at least one third. Where indicated in table legends, relative risks (RRs) are also presented, calculated using a multivariable model adjusted for prespecified risk factors for hypoglycaemia.

We anticipated that these data might yield a number of “possible” doses in which the 95% CI for the odds of hypoglycaemia was significantly lower than the placebo dose (i.e., P odds of hypoglycaemia < 0.05). Therefore, we also undertook a complementary analysis of limitations at each dose level.

The odds of at least one limitation for each cumulative dose arm relative to the placebo dose was estimated (with 95% CIs) and plotted, and the likelihood that this estimate differed from the placebo arm was reported. Additionally, a limitation score, comprising the sum of weights assigned to the predetermined limitations (maximum score 18.5), was summarised for each cumulative dose arm (median ± 95% CI, Mid-P method) and plotted. We anticipated that about 5% of untreated babies might experience hyperglycaemia, 20% late hypoglycaemia, and 25% delayed feeding. We anticipated that seizures due to hypoglycaemia and infant death were extremely unlikely.

Analysis was performed using SAS (v9.3 SAS Institute) on an intention-to-treat basis. All tests were two-tailed, and *p* < 0.05 was considered statistically significant. Since these were exploratory analyses, no adjustment for multiplicity was performed. Data are presented as number (%), median (range), mean difference (95% CI), and RR (95% CIs) as appropriate.

## Results

### Baseline Details

A total of 416 babies were randomised between August 3, 2013, and November 13, 2014 (Fig 1).

Demographic and baseline characteristics were similar for all randomisation groups (Table 1). The median (range) birthweight was 3,190 (2,200, 5,255) g and gestational age 38 (35, 42) wk; 301/415 babies (73%) were infants of mothers with diabetes, and 199/415 (48%) were born by caesarean delivery. Primary risk factors for hypoglycaemia were similar across all treatment groups. A similar proportion of mothers in each group were uncertain as to the gel type the baby received (163/257 [63%] in those randomised to dextrose gel versus 77/126 [61%] in those randomised to placebo) or thought the baby had received dextrose gel (67/277 [24%] randomised to dextrose versus 34/126 [25%] randomised to placebo), demonstrating effective masking.

**Table 1:** Baseline characteristics of mothers and babies allocated to each dosage group.

	Single Dose			Multiple Dose				
	Placebo	Dextrose 0.5 ml/kg	Dextrose 1 ml/kg	Placebo	Dextrose 0.5 ml/kg x 4	Dextrose 1 ml/kg x 1, 0.5 ml/kg x 3	Any Dose of Placebo Gel	Any Dose of Dextrose Gel
**Mothers (*n* = 403** * **)**								
**Number**	70	66	71	68	68	70	138	275
Maternal Age (y)	33 (5)	33 (5)	33 (5)	32 (6)	32 (5)	32 (5)	32 (6)	32 (5)
Parity	2 (1–8)	2 (0–7)	1 (1–5)	2 (0–5)	2 (1–10)	2 (1–8)	2 (0–8)	2 (0–10)
Weight at Booking (kg)	77 (23)	79 (23)	76 (23)	74 (21)	75 (24)	77 (26)	76 (22)	77 (24)
Diabetic	51 (73)	49 (74)	51 (72)	49 (72)	51 (75)	50 (71)	100 (72)	201 (73)
Type 1 Diabetes	5 (10)	3 (6)	2 (4)	2 (4)	3 (6)	3 (6)	7 (7)	11 (5)
Type 2 Diabetes	4 (8)	3 (6)	7 (14)	3 (6)	4 (8)	4 (8)	7 (7)	18 (9)
Gestational Diabetes	42 (82)	43 (88)	42 (82)	44 (90)	44 (86)	43 (86)	86 (86)	172 (86)
Insulin Therapy	37 (73)	33 (67)	37 (73)	36 (73)	40 (78)	31 (62)	73 (73)	141 (70)
Pre-eclampsia	4 (6)	3 (5)	1 (1)	2 (3)	1 (1)	4 (6)	6 (4)	9 (3)
Hypertension	3 (4)	6 (9)	8 (11)	7 (10)	4 (6)	14 (20)	10 (7)	32 (12)
Prioritised Ethnicity								
Māori	9 (13)	6 (9)	5 (7)	10 (15)	8 (12)	4 (6)	9 (14)	23 (8)
Pacific	9 (13)	13 (20)	12 (17)	11 (16)	9 (13)	13 (19)	20 (14)	47 (17)
Chinese	5 (7)	7 (11)	8 (11)	10 (15)	8 (12)	6 (9)	1 (11)	29 (11)
Indian	12 (17)	6 (9)	9 (13)	13 (19)	15 (22)	7 (10)	25 (18)	37 (13)
Other	15 (21)	19 (29)	11 (15)	15 (22)	10 (15)	14 (20)	30 (22)	54 (20)
NZ European	20 (29)	15 (23)	26 (37)	9 (13)	18 (26)	26 (37)	29 (21)	85 (31)
**Babies (*n* = 415)**								
**Number**	70	66	73	68	68	70	138	277
Female	30 (43)	36 (55)	42 (58)	33 (49)	34 (50)	27 (39)	63 (46)	139 (50)
Birthweight (g)	3,210 (653)	3,265 (627)	3,251 (611)	3,288 (613)	3,231 (580)	3,229 (621)	3,248 (633)	3,244 (607)
Birthweight Z Score	0.07 (1.33)	0.28 (1.30)	0.20 (1.31)	0.18 (1.32)	0.09 (1.32)	0.03 (1.20)	0 (1)	0 (1)
Gestation (wk)	38 (1)	38 (1)	38 (1)	38 (1)	38 (1)	38 (1)	38 (1)	38 (1)
Singleton Birth	65 (93)	61 (92)	65 (89)	63 (93)	65 (97)	65 (93)	128 (93)	256 (92)
Caesarean Birth	36 (51)	35 (53)	33 (45)	36 (53)	27 (40)	32 (46)	72 (52)	127 (46)
Apgar Score of <5 at 5 min	2 (3)	0 (0)	0 (0)	0 (0)	0 (0)	0 (0)	2 (1)	0 (0)
Primary Risk Factor for Hypoglycaemia								
Infant of Mother with Diabetes	51 (73)	49 (74)	51 (70)	49 (72)	51 (75)	50 (71)	100 (72)	201 (73)
Late Preterm	3 (4)	5 (8)	7 (10)	3 (4)	3 (4)	6 (9)	6 (4)	21 (8)
Small	10 (14)	7 (11)	8 (11)	8 (12)	7 (10)	9 (13)	18 (13)	31 (11)
Large	6 (9)	5 (8)	7 (10)	8 (12)	7 (10)	5 (7)	14 (10)	24 (9)
Babies with Two Risk Factors	7 (21)	3 (8)	10 (14)	17 (26)	9 (12)	2 (6)	4 (6)	6 (9)
Babies with Three Risk Factors	0 (0)	1 (3)	1 (1)	0 (0)	0 (0)	0 (0)	0 (0)	0 (0)

Data are *n* (%), mean (standard deviation [SD]), or median (range).

* There are 12 mothers of twins, of whom 10 appear in more than one column because each twin was assigned to a different treatment group.

The overall incidence of hypoglycaemia was 186/415 (45%, 95% CI 40%–50%), and 32/415 babies (8%, 95% CI 5.5%–10.7%) were admitted to NICU, of whom most (23/415, 6%, 95% CI 3.7%–8.2%) were admitted for hypoglycaemia. For those babies who became hypoglycaemic, the median (range) age at first detection was 2.3 (1.1, 44.5) h. Formula was given to 232/415 (56%, 95% CI 51%–61%) during hospital admission, with the most common indications being medical intervention for hypoglycaemia (93/232, 40%, 95% CI 34%–47%) and maternal choice (73/232, 31%, 95% CI 26%–38%). At discharge from hospital, 279/407 (69%, 95% CI 64%–73%) babies were fully or exclusively breastfeeding.

### Compliance

There was no difference between placebo and dextrose gel groups in timing of gel administration or in volume of gel administered (Table 2). Thirty babies did not receive all doses of gel according to protocol, including 13 who were withdrawn from the trial prior to completing all doses, 7 with missed doses, and 8 who received an incorrect volume. These protocol deviations in gel administration occurred with similar frequency for babies allocated to dextrose gel and placebo (21/277, 8% versus 9/138, 7%). Only 1/209 babies (<1%) allocated to a single dose did not receive all allocated gel, compared to 26/206 babies (13%) allocated to multiple doses (RR = 0.04, 95% CI 0.01–0.28, *p* = 0.0013).

**Table 2:** Details of study gel and supplementary dextrose administration for each dosage group.

	Single Dose			Multiple Dose					
	Placebo	Dextrose 0.5 ml/kg	Dextrose 1 ml/kg	Placebo	Dextrose 0.5 ml/kg x 4	Dextrose 1 ml/kg x 1, 0.5 ml/kg x 3	Any Dose of Placebo	Any Dose of Dextrose	Relative Risk or Mean Difference (95% CI), *p*
Number of Babies Allocated to Study Gel	70	66	73	68	68	70	138	277	
**Study Gel**									
Age at First Dose (h)	1.0 (0.5–1.3)	1.0 (0.4–1.3)	1.0 (0.5–1.7)	1.0 (0.7–1.2)	1.0 (0.3–1.2)	1.0 (0.4–1.6)	1.0 (0.5–1.3)	1.0 (0.3–1.7)	−0.01 (−0.04 to 0.02), *p* = 0.70
Age at Second Dose (h)	–	–	–	4.6 (2.8–9.4)	4.6 (2.4–7.2)	4.5 (1.8–8.8)	4.6 (2.8–9.4)	4.5 (1.8–8.8)	−0.19 (-0.53 to 0.15), *p* = 0.27
Age at Third Dose (h)	–	–	–	7.8 (5.8–14.1)	7.9 (5.9–10.5)	7.9 (3.6–12.3)	7.8 (5.8–14.1)	7.9 (3.6–12.3)	−0.10 (−0.49 to 0.28), *p* = 0.59
Age at Fourth Dose (h)	–	–	–	11.1 (8.1–18.9)	11.2 (9.1–16.4)	11.1 (8.4–15.5)	11.1 (8.1–18.9)	11.2 (8.4–16.4)	−0.17 (−0.70 to 0.36), *p* = 0.52
Total Volume of Study Gel (ml/kg)*	1.0 (0.2–1.0)	0.5 (0.5–0.6)	1.0 (1.0–1.1)	2.0 (0.5–3.1)	2.0 (0.0–2.6)	2.5 (1.0–2.6)	1.0 (0.2–3.1)	1.0 (0.0–2.6)	−0.00 (−0.16 to 0.16), *p* = 0.97
Total Dose of Dextrose as Study Gel (g/kg)	0	0.2 (0.2–0.2)	0.4 (0.4–0.5)	0	0.8 (0.0–1.0)	1.0 (0.4–1.0)	0	0.4 (0.0–1.0)	
**Supplementary Dextrose**									
Received Any Supplementary Dextrose *n* (%)	25 (36)	15 (23)	16 (22)	17 (25)	19 (28)	17 (24)	42 (30)	67 (24)	0.79 (0.57–1.10), *p* = 0.17
Total Dose of Supplementary Dextrose (g/kg)	0.2 (0.2–17.8)	0.4 (0.2–14.6)	0.2 (0.2–3.8)	0.4 (0.2–32.0)	0.4 (0.2–9.0)	0.2 (0.2–11.6)	0.4 (0.2–32.0)	0.4 (0.2–14.6)	−1.16 (−3.17 to 0.85), *p* = 0.25
Received Dextrose Gel for Treatment *n* (%)	22 (31)	13 (20)	16 (22)	17 (25)	17 (25)	16 (23)	39 (28)	62 (22)	0.79 (0.56–1.12), *p* = 0.18
Total Dose of Treatment Gel (g/kg)	0.2 (0.2–1.0)	0.4 (0.2–0.8)	0.2 (0.2–0.8)	0.4 (0.2–0.6)	0.2 (0.2–0.8)	0.2 (0.2–0.6)	0.2 (0.2–1.0)	0.2 (0.2–0.8)	−0.004 (−0.08 to 0.07), *p* = 0.92
Received Any Intravenous Dextrose (Bolus or Infusion) *n* (%)	7 (10)	3 (5)	2 (3)	4 (6)	5 (7)	4(6)	11 (8)	14 (5)	0.63 (0.30–1.36), *p* = 0.24
Total Dose of Intravenous Dextrose (Bolus or Infusion) per Baby (g/kg)	4.2 (0.3–17.6)	6.0 (1.9–14.6)	2.3 (1.2–3.4)	6.1 (4.1–31.5)	4.4 (2.08–8.78)	5.7 (1.3–11.4)	5.0 (0.3–31.5)	4.3 (1.2–14.6)	−3.38 (−10.01 to 3.24), *p* = 0.29
**Total Dextrose Dose (Prophylaxis Plus Supplementary) (g/kg)**	0.0 (0.0–17.8)	0.2 (0.2–14.8)	0.4 (0.4–4.2)	0.0 (0.0–32.0)	0.8 (0.0–9.8)	1.0 (0.4–12.6)	0.0 (0.0–32.0)	0.8 (0.0–14.8)	0.13 (−0.50 to 0.75), *p* = 0.69

Data are median (range), number (%), RR (95% CI), or mean difference (95% CI) for comparison between any dose of placebo and any dose of dextrose.

* Did not receive all allocated study gel: single dose placebo, 1; multiple dose placebo, 7; multiple dose dextrose 0.5 ml/kg x 4, 10; multiple dose dextrose 1 ml/kg x 1 0.5 ml/kg x 3, 8.

Twenty-four babies (24/415, 6%) were withdrawn from the trial after randomisation (Fig 1), although consent was given to obtain outcome data from the clinical records for these babies. Withdrawal rates were similar in babies randomised to dextrose gel and placebo (17/277 [6%] versus 7/138 [5%], *p* = 0.60) but were higher in babies randomised to multiple doses than in those randomised to a single dose of gel (17/206 [8%] versus 7/209 [3%], RR 1.05, 95% CI 1.00–1.11, *p* = 0.034). The most common reasons for withdrawal were parental concern about blood sampling (despite this not being determined by the trial protocol) and clinician uncertainty about the trial protocol.

### Efficacy of Different Doses

**Fig 2:**
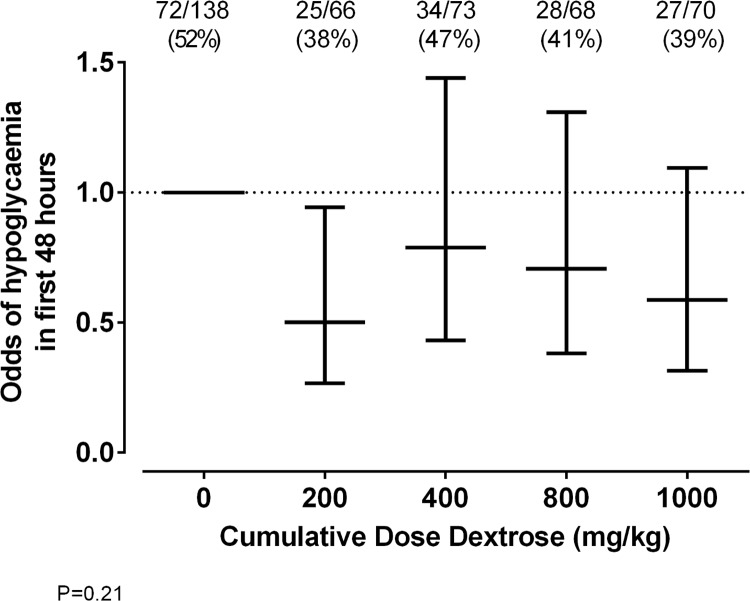
Odds of hypoglycaemia for each cumulative dose of prophylactic dextrose gel. Odds ratios of blood glucose concentration < 2.6 mM for each cumulative dose of prophylactic dextrose gel, where 0 mg/kg is placebo, 200 mg/kg is 0.5 ml/kg dextrose once, 400 mg/kg is 1 ml/kg once, 800 mg/kg is 0.5 ml/kg for four doses, and 1,000 mg/kg is 1 ml/kg once followed by 0.5 ml/kg for a further three doses. Data are odds ratios +/− 95% CI adjusted for prespecified potential confounders (sex, gestational age, and delivery mode), and the numerals above the figure are the number (%) of babies who experienced hypoglycaemia (blood glucose concentration < 2.6 mM) in each group.

When cumulative doses of dextrose gel were plotted against the odds of developing hypoglycaemia, with adjustment for sex, gestational age, and mode of birth (Fig 2), the odds of hypoglycaemia were not significantly lower when all dose regimes of dextrose gel were compared against placebo gel (*p* = 0.21). However, the 95% CI for the 200 mg/kg dose relative to placebo did not include unity.

**Fig 3:**
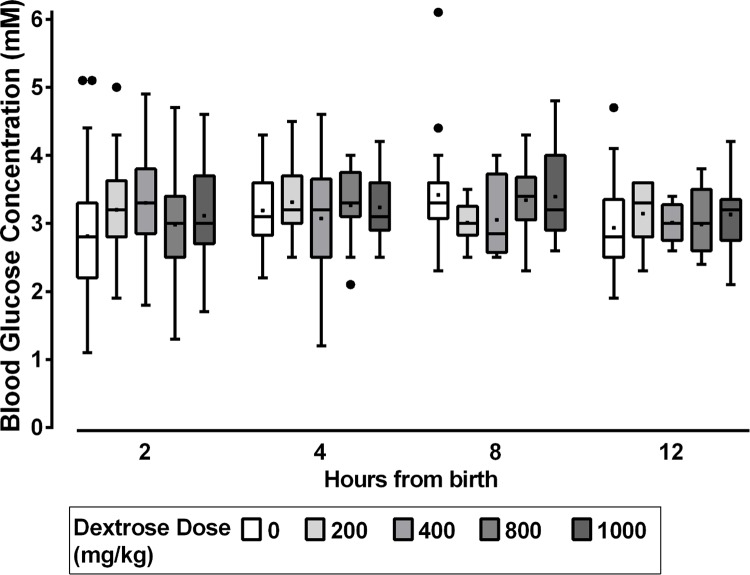
Median blood glucose concentration at four time intervals for each dose regime of dextrose gel. Boxplots for blood glucose concentration (mM) at time = 2, 4, 8, and 12 h ± 30 min, for each cumulative dose of prophylactic dextrose gel, where 0 mg/kg is placebo, 200 mg/kg is 0.5 ml/kg dextrose once, 400 mg/kg is 1 ml/kg once, 800 mg/kg is 0.5 ml/kg for four doses, and 1,000 mg/kg is 1 ml/kg once followed by 0.5 ml/kg for a further three doses. The box represents 25th to 75th percentiles. The horizontal bar within the box is the median, and the solid dot within the box is the mean. The whiskers are 1.5 (interquartile range [IQR]) above and below the 25th and 75th percentile. Solid dots beyond the whiskers represent outliers.

In post hoc exploratory analyses, there was no difference in median blood glucose concentration between dose regimes (Fig 3). Amongst babies randomised to multiple doses of dextrose gel who became hypoglycaemic, 56/88 (64%) had done so before the time that they would have completed their allocated four doses of gel.

### Overall Efficacy

Babies randomised to any dose of dextrose gel were less likely to develop hypoglycaemia than those randomised to placebo (RR 0.79, 95% CI 0.64–0.98, *p* = 0.03; number needed to treat = 10, 95% CI 5–115, Table 3). They also developed hypoglycaemia later (dextrose 3.7 [1.1–44.5] h, placebo 2.1 [1.5–43.8] h, *p* = 0.03). However, the lowest blood glucose concentration in those who did experience hypoglycaemia was similar for babies randomised to dextrose gel or to placebo (2.3 [0.6–2.5] mM versus 2.1 [1.1–2.5] mM, mean difference 0.08 mM, 95% CI −0.02 to 0.18 mM, *p* = 0.13), as was the number of blood glucose measurements < 2.6 mM in the first 48 h (mean [standard deviation (SD)] 1.8 [1.1] versus 2.0 [1.2], mean difference −0.19, 95% CI −0.54 to 0.16 mM, *p* = 0.29). One quarter of the babies (109/415, 26%) received supplementary dextrose in addition to study gel, either as open label 40% dextrose gel or intravenous dextrose, with similar rates in dextrose and placebo gel groups (67/277 [24%] versus 42/138 [30%], RR 0.79, 95% CI 0.57–1.10, *p* = 0.17, Table 2).

**Table 3:** Primary and secondary outcomes for each dosage group.

	Single Dose					Multiple Dose								
	Placebo	Dextrose 0.5 ml/kg		Dextrose 1 ml/kg		Placebo	Dextrose 0.5 ml/kg x 4		Dextrose 1 ml/kg x 1, 0.5 ml/kg x 3		Any Dose of Placebo	Any Dose of Dextrose		Total
	*n* (%) or Median (Range)	*n* (%) or Median (Range)	RR or Mean Difference (95% CI), *p*	*n* (%) or Median (Range)	RR or Mean Difference (95% CI), *p*	*n* (%) or Median (Range)	*n* (%) or Median (Range)	RR or Mean Difference (95% CI), *p*	*n* (%) or Median (Range)	RR or Mean Difference (95% CI), *p*	*n* (%) or Median (Range)	*n* (%) or Median (Range)	RR or Mean Difference (95% CI), *p*	*n* (%) or Median (Range)
Number of Babies	70	66		73		68	68		70		138	277		415
Hypoglycaemia	39 (56)	25 (38)	0.68 (0.47–0.99), *p* = 0.04	34 (47)	0.84 (0.61–1.15), *p* = 0.28	33 (49)	28 (41)	0.85 (0.58–1.23), *p* = 0.39	27 (39)	0.79 (0.54–1.17), *p* = 0.24	72 (52)	114 (41)	RR: 0.79 (0.64–0.98), *p* = 0.03	186 (45)
Age Hypoglycaemia First Detected (h)	2.1 (1.5–12.1)	4.7 (1.6–38.0)	3.74 (−0.37 to 7.85), *p* = 0.07	4.1 (1.3–44.4)	2.04 (−0.82 to 4.90), *p* = 0.25	2.1 (1.5–43.8)	2.2 (1.1–11.8)	−1.75 (−5.41 to 1.90), *p* = 0.79	3.0 (1.9–44.5)	0.68 (−4.46 to 5.81), *p* = 0.04	2.1 (1.5–43.8)	3.7 (1.1–44.5)	1.20 (−1.07 to 3.48), *p* = 0.03	2.3 (1.1–44.5)
Admission to NICU	10 (14)	3 (5)	0.32 (0.09–1.11), *p* = 0.07	3 (4)	0.29 (0.08–1.00), *p* = 0.05	4 (6)	8 (12)	2.00 (0.63–6.33), *p* = 0.24	4 (6)	0.97 (0.25–3.73), *p* = 0.97	14 (10)	18 (7)	0.64 (0.33–1.25), *p* = 0.19	32 (8)
Admission to NICU for Hypoglycaemia	9 (13)	1 (2)	0.12 (0.02–0.90), *p* = 0.04	3 (4)	0.32 (0.09–1.13), *p* = 0.08	3 (4)	5 (7)	1.67 (0.41–6.70), *p* = 0.47	2 (3)	0.65 (0.11–3.76), *p* = 0.63	12 (9)	11 (4)	0.46 (0.21–1.01), *p* = 0.05	23 (6)
Late Hypoglycaemia	1 (1)	3 (5)	3.18 (0.34–29.83), *p* = 0.31	4 (5)	3.84 (0.44–33.48), *p* = 0.22	2 (3)	0	NC	2 (3)	0.97 (0.14–6.70), *p* = 0.98	3 (2)	9 (3)	1.49 (0.41–5.43), *p* = 0.54	12 (3)
Breastfeeding at Discharge (Full or Exclusive)	48/69 (70)	43/66 (65)	0.94 (0.74–1.19), *p* = 0.59	44/73 (60)	0.87 (0.68–1.10), *p* = 0.25	45/66 (68)	49/64 (77)	1.12 (0.91–1.39), *p* = 0.29	50/69 (72)	1.06 (0.85–1.32), *p* = 0.59	93/135 (69)	186/272 (68)	0.99 (0.86–1.14), *p* = 0.92	279/407
Received Any Formula Prior to Discharge	42/69 (61)	41/66 (62)	1.02 (0.78–1.33), *p* = 0.88	45/73 (62)	1.01 (0.78–1.32), *p* = 0.92	40/66 (61)	30/64 (47)	0.77 (0.56–1.07), *p* = 0.12	34/69 (49)	0.81 (0.60–1.11), *p* = 0.19	82/135 (61)	150/272 (55)	0.91 (0.76–1.08), *p* = 0.27	232
Number of Formula Feeds per Formula Fed Baby	18 (1–56)	10 (1–45)	−6.00 (−11.58 to 0.41), *p* = 0.036	15 (1–66)	0.40 (−6.29 to 7.10), *p* = 0.91	11 (1–52)	10 (2–78)	−0.57 (−8.12 to −6.98), *p* = 0.88	18 (1–56)	4.23 (−2.64 to 11.10), *p* = 0.22	12 (1–56)	12 (1–78)	−0.48 (−4.48 to 3.52), *p* = 0.81	12 (1–78)
Volume of Formula (ml/kg/feed)	3.8 (1.2–11.5)	3.1 (1.5–9.3)	−0.08 (−0.96 to 0.81), *p* = 0.86	2.9 (0.4–9.2)	−0.82 (−1.58 to −0.05), *p* = 0.037	3.2 (0.4–10.7)	3.4 (0.4–11.2)	−0.15 (−1.02 to 0.72), *p* = 0.73	3.9 (0.7–9.0)	0.42 (−0.42 to −1.26), *p* = 0.32	3.5 (0.4–11.5)	3.3 (0.4–11.2)	−0.17 (−0.68 to 0.34), *p* = 0.51	3.4 (0.4–11.5)
Breastfeeding on Day 3 (Full or Exclusive)	34/69 (49)	38/64 (59)	1.21 (0.88–1.65), *p* = 0.24	38/73 (52)	1.06 (0.76–1.46), *p* = 0.74	35/66 (53)	45/64 (70)	1.33 (1.00–1.75), *p* = 0.046	43/69 (62)	1.18 (0.88–1.57), *p* = 0.28	69/135 (51)	164/270 (61)	1.19 (0.98–1.44), *p* = 0.08	233/405
Delayed Feeding	35/69 (51)	26/64 (41)	0.80 (0.55–1.17), *p* = 0.25	35/73 (48)	0.95 (0.68–1.32), *p* = 0.74	31/66 (47)	19/64 (30)	0.63 (0.40–1.00), *p* = 0.049	26/69 (38)	0.80 (0.54–1.19), *p* = 0.28	66/135 (49)	106/270 (39)	0.80 (0.64–1.01), *p* = 0.06	172/405 (42)
Receiving Some Formula at 6 wk	32/66 (48)	28/61 (46)	0.95 (0.65–1.37), *p* = 0.77	32/70 (46)	0.94 (0.66–1.35), *p* = 0.75	28/63 (44)	22/61 (36)	0.81 (0.53–1.25), *p* = 0.34	29/65 (45)	1.00 (0.68–1.48), *p* = 0.98	60/129 (47)	111/257 (43)	0.93 (0.74–1.17), *p* = 0.53	171/386 (44)
Parental Satisfaction	65/68 (96)	61/66 (92)	0.97 (0.89–1.05), *p* = 0.44	68/73 (93)	0.97 (0.90–1.06), *p* = 0.53	59/64 (92)	55/63 (87)	0.95 (0.84–1.07), *p* = 0.37	58/68 (85)	0.93 (0.82–1.05), *p* = 0.21	124/132 (94)	242/270 (90)	0.95 (0.90–1.01), *p* = 0.12	366/402 (91)

Data are *n* (%), median (range), RR (95% CI), or mean difference (95% CI) for comparison with relevant placebo gel group. NC, not calculable. Missing data: Feeding method at discharge, 8; feeding method on day 3, 10; feeding method at 6 wk, 29; parental satisfaction, 13.

There was no difference between dextrose and placebo groups in the rate of admission to a NICU (Table 3), although admission to a NICU for hypoglycaemia tended to be less common in babies randomised to dextrose gel (RR 0.46, 95% CI 0.21–1.01, *p* = 0.05). Rates of breastfeeding were similar in both groups at discharge (*p* = 0.92), on day 3 (*p* = 0.08), and at 6 wk (*p* = 0.53) (Table 3). Parental satisfaction did not differ for babies who received single doses rather than multiple doses (RR 1.06, 95% CI 1.00–1.13, *p* = 0.06) or for babies who received dextrose or placebo gel (RR 0.95, 95% CI 0.90–1.01, *p* = 0.12).

In post hoc subgroup analysis, the effect of dextrose gel on the incidence of hypoglycaemia was similar in babies with different primary risk factors.

### Limitations

Overall, gel was well tolerated, with 918 of 1,030 doses (89%) associated with no spill or a small spill, 33 (3%) with a moderate, and 13 (1%) with a large spill. At least one moderate or large spill was more common in babies receiving multiple doses than after single doses (RR 7.94, 95% CI 2.85–22.09, *p* < 0.001) but was not different between babies receiving dextrose and placebo gel (RR 1.09, 95% CI 0.55–2.17, *p* = 0.80).

Most doses took 5 to 10 min to administer. Taking longer than 5 min to administer a dose was more common for multiple doses than for single doses (RR 1.08, 95% CI 1.03–1.14, *p* = 0.0036) but was similar for dextrose gel and placebo gel administration (RR 1.05, 95% CI 0.99–1.11, *p* = 0.13).

Similarly, parents reported more messiness with multiple doses than with single doses of gel (RR 7.07, 95% CI 2.14–23.33, *p* = 0.0013) but no differences between dextrose and placebo gel (RR 1.00, 95% CI 0.44–2.29, *p* = 0.99). Most parents found the gel acceptable (364/402, 91%), with no differences between multiple and single doses or between dextrose and placebo gel.

No babies met the criteria for hyperglycaemia. There were no differences between treatment groups in the incidence of late hypoglycaemia or delayed feeding (Table 4).

**Table 4:** Limitation scores.

	Limitation Weighting	Single Dose			Multiple Dose								
		Placebo	Dextrose 0.5 ml/kg	Dextrose 1 ml/kg	Placebo	Dextrose 0.5 ml/kg x 4	Dextrose 1 ml/kg x 1, 0.5 ml/kg x 3	Any Dose of placebo	Any Dose of Dextrose		Any Single Dose	Any Multiple Dose	
										RR (95% CI), *p*			RR (95% CI), *p*
Number of Babies		70	66	73	68	68	70	138	277		209	206	
**Tolerance**													
No Spill		0	59/70 (84)	53/66 (80%)	46/73 (63)	40/68 (59)	34/66 (52)	45/70 (64)	99/138 (72)	156/275 (57)		158/209 (76)	97/204 (48)
Small Spill	0	9/70 (13)	13/66 (20)	25/73 (34)	19/68 (23)	23/66 (35)	34/70 (49)	28/138 (20)	95/275 (35)		47/209 (22)	76/204 (37)	
Moderate Spill	1	2/70 (3)	0	2/73 (3)	8/68 (12)	5/66 (8)	9/70 (13)	10/138 (7)	16/275 (6)		4/209 (2)	22/204 (11)	
Large Spill	2	0/70 (0)	0	0	1/68 (1)	6/66 (9)	2/70 (3)	1/138 (1)	8/275 (3)		0	9/204 (4)	
Tolerance Score > 0		2/70 (3)	0/66 (0)	2/73 (3)	9/68 (13)	11/66 (17)	11/70 (16)	11/138 (8)	24/275 (9)	1.09 (0.55–2.17), *p* = 0.80	4/209 (2)	31/204 (15)	7.94 (2.85–22.09), *p* < 0.001
**Time to Administer**													
<5 min	0	11/70 (16)	7/66 (11)	3/73 (4)	2/68(3)	4/66 (6)	0/70	13/138 (9)	14/275 (5)		21/209 (10)	6/204 (3)	
5–10 min	1	53/70 (76)	57/66 (86)	53/73 (73)	55/68 (81)	52/66 (79)	47/70 (67)	108/138 (78)	209/275 (76)		163/209 (78)	154/204 (75)	
>10 min	2	6/70 (9)	2/66 (3)	17/73 (23)	11/68 (16)	10/66 (15)	23/70 (33)	17/138 (12)	52/275 (19)		25/209 (12)	44/204 (22)	
Time Score > 0		59/70 (84)	59/66 (89)	70/73 (96)	66/68 (97)	62/66 (94)	70/70 (100)	125/138 (91)	261/275 (95)	1.05 (0.99–1.11), *p* = 0.13	188/209 (90)	198/204 (97)	1.08 (1.03–1.14), *p* = 0.0036
**Messiness**													
No	0	67/67 (100)	62/63 (98)	68/70 (97)	58/66 (88)	60/63 (95)	59/69 (86)	125/133 (94)	249/265 (94)		197/200 (98)	177/198 (89)	
Yes	0.5	0	1/63 (2)	2/70 (3)	8/66 (12)	3/63 (5)	10/69 (14)	8/133 (6)	16/265 (6)	1.00 (0.44–2.29), *p* = 0.99	3/200 (2)	21/198 (11)	7.07 (2.14–23.33), *p* = 0.0013
Hyperglycaemia	6	0	0	0	0	0	0						
Late Hypoglycaemia	6	1/70 (1)	3/66 (5)	4/73 (5)	2/68 (3)	0/68 (0)	2/70 (3)	3/138 (2)	9/277 (3)	1.49 (0.41–5.43), *p* = 0.54	8/209 (4)	4/206 (2)	0.51 (0.16–1.66), *p* = 0.26
Delayed Feeding	2	35/69 (51)	26/64 (41)	35/73 (48)	31/66 (47)	19/64 (30)	26/69 (38)	66/135 (49)	106/270 (39)	0.80 (0.64–1.01), *p* = 0.06	96/206 (47)	76/199 (38)	0.82 (0.65–1.03), *p* = 0.09
**Acceptability**													
Acceptable	0	65/68 (96)	61/66 (92)	68/73 (93)	59/64 (92)	55/63 (87)	58/68 (85)	124/132 (93)	242/270 (90)		194/207 (94)	172/195 (88)	
Some Inconvenience	0	2/68 (3)	4/66 (6)	3/73 (4)	3/64 (5)	7/63 (11)	9/68 (13)	5/132 (4)	23/270 (9)		9/207 (4)	19/195 (10)	
Major Inconvenience	1	0/68 (0)	0/66 (0)	1/73 (1)	1/64 (2)	1/63 (2)	0/68 (0)	1/132 (1)	2/270 (1)		1/207 (<1)	2/195 (1)	
Unacceptable	2	1/68 (1)	1/66 (2)	1/73 (1)	1/64 (2)	0/63 (0)	1/68 (1)	2/132 (2)	3/270 (1)		3/207 (1)	2/195 (1)	
Acceptability Score > 0		1/68 (1)	1/66 (2)	2/73 (3)	2/64 (3)	1/63 (2)	1/68 (1)	3/132 (2)	5/270 (2)	0.81 (0.20–3.36), *p* = 0.78	4/207 (2)	4/195 (2)	1.06 (0.27–4.19), *p* = 0.93
**Total Limitation Score**		2 (0–7)	1 (0–9)	3 (0–10)	2 (1–10)	2 (0–5)	2 (1–10)	2 (0–10)	2 (0–10)	0.01 (−0.32 to 0.34), *p* = 0.94	2 (0–10)	2 (0–10)	0.10 (−0.21 to 0.41), *p* = 0.53
**Total Limitation Score > 0**		66/70 (94)	61/66 (92)	72/73 (99)	68/68 (100)	63/66 (95)	70/70 (100)	134/138 (97)	266/275 (97)	RR: 1.00 (0.96–1.03), *p* = 0.83	199/209 (95)	201/204 (99)	RR: 1.03 (1.00–1.07), *p* = 0.053

Data are *n* (%), median (range) or RR (95% CI) for comparison between adjacent columns. Limitation weightings were arbitrarily assigned in advance, based on consensus of clinical importance. Small spill = (few drops), moderate spill = (half of volume administered), large spill = (all of volume administered), messiness (parental report), acceptability (parental report), delayed feeding = failure to establish breast feeding without supplementation by the end of day 3.

Total limitations scores were similar in all treatment groups. However, more babies in the multiple dose group than in the single dose group experienced at least one limitation (score > 0, RR 1.03, 95% CI 1.00–1.07, *p* = 0.05), although this was similar in dextrose and placebo gel groups (RR 1.00, 95% CI 0.96–1.03, *p* = 0.83).

### Adverse Effects

One baby developed seizures, without concurrent hypoglycaemia, that were not considered to be related to the intervention. There were no neonatal or infant deaths. No babies developed hyperglycaemia or systemic sepsis or had a first episode of hypoglycaemia after 48 h. Delayed feeding occurred in 170/405 (42%, 95% CI 37%–47%) babies, and late hypoglycaemia in 14/415 (3.4%, 95% CI 2.0%–5.6%), with similar rates in all treatment groups (Table 3).

## Discussion

Our findings show that the most effective and well-tolerated dose of prophylactic oral dextrose gel to reduce the incidence of neonatal hypoglycaemia in babies born at risk but without indication for a NICU admission is 200 mg/kg (0.5 ml/kg of 40% oral dextrose gel). Further, the intervention was easy to administer, well tolerated, acceptable to parents, and not associated with any adverse outcomes. Neonatal hypoglycaemia is a common problem, occurring in up to 15% of newborn babies and in 50% of those born at risk [1]. Management commonly includes supplementary feeds with formula milk and/or separation of mother and baby for admission to a NICU for more invasive management with intravenous dextrose. The use of formula milk is associated with decreased breastfeeding rates [10], and admission to a NICU separates mother and baby, making breastfeeding establishment more difficult as well as increasing health care costs. Other than feeding early [5], there are no effective interventions for prophylaxis of neonatal hypoglycaemia in babies at risk. This trial is the first to demonstrate that oral dextrose gel reduces the incidence of neonatal hypoglycaemia.

Neonatal hypoglycaemia occurs most frequently in the first 24 h after birth, with lower blood glucose concentrations of ≤2 mM occurring most often within the first 12 h [1]. Babies at risk of neonatal hypoglycaemia commonly receive repeated feeds of supplemental formula milk to manage low blood glucose measurements while maternal lactation is established [6,10]. Therefore, we decided to investigate the effect of a prophylactic regime of multiple doses of dextrose given within the first 12 h following birth. As with the single dose regime, we considered that both a standard and a higher initial dose might be of benefit.

Perhaps surprisingly, there was no evidence of a dose-response effect, with all dose regimes having similar efficacy and resulting in similar median blood glucose concentrations. However, the diagnosis of hypoglycaemia was later in babies randomised to dextrose gel, although the incidence of late hypoglycaemia was unchanged, suggesting that the main effect of dextrose gel may be in reducing the incidence of early hypoglycaemia. This is consistent with our finding that two-thirds of babies randomised to multiple doses who developed hypoglycaemia did so before the time that they would have received all doses. These babies had already met the primary outcome, and therefore, any benefit of subsequent doses in maintaining blood glucose concentrations would not be captured in this analysis. For the same reason, the effective sample size was less than the number randomized in the multiple dose groups, and it is therefore possible that the study was inadequately powered to detect differences between single and multiple dose groups. Within the single dose groups, there was no indication that higher doses might have been effective, with the proportion of hypoglycaemic babies in the 1 ml/kg dose group being closer to that in the placebo group than in the 0.5 ml/kg dose group.

Each dose of dextrose gel was followed by a breast feed. We anticipated that the dextrose gel would be rapidly absorbed into the buccal mucosa but alone would not be adequate to maintain blood glucose concentrations for the length of the period between feeds. Although early colostrum contains few calories, it contains many other factors that are important for early neonatal health, including metabolic regulation during the transition [11,12], and we considered it a priority to encourage early establishment of breastfeeding, with health benefits for both mother and baby [13,14]. It was also possible that the gel might stimulate insulin production. Although there is uncertainty whether increased blood glucose concentration in the early neonatal period does induce an increase in insulin production [15], transient neonatal hyperinsulinism is the likely mechanism underlying most transient neonatal hypoglycaemia [16].

It should be noted that we measured whole-blood glucose concentrations using the i-STAT portable clinical analyser, which utilises the glucose oxidase method and does not adjust the results to plasma glucose concentrations. Screening of babies at risk for neonatal hypoglycaemia is commonly performed using whole blood and bedside analysers, rather than plasma, because of the requirement for immediate results and the risk of glycolysis in specimens sent to the lab [2,6]. Plasma glucose concentrations are approximately 10% to 18% higher than whole-blood concentrations because of the higher water content of plasma [2]. Ten percent of blood tests in this trial were analysed using the blood gas analyser in the NICU, usually after the baby had been diagnosed with hypoglycaemia and admitted to the NICU. Good reliability between the i-STAT and blood gas analysers in measuring blood glucose concentrations in this population has previously been reported [17].

The eligibility criteria for this study were intended to select babies who would not need NICU admission for other reasons and were therefore most likely to benefit if hypoglycaemia could be avoided. Although admission to a NICU for hypoglycaemia appeared to be less common in babies allocated to dextrose gel, overall admission rates were similar in both groups. However, this dose-finding trial was not powered to detect a reduction in admission to NICUs or later neurodevelopmental outcomes, and therefore, a larger trial is needed to determine the effect of prophylactic dextrose gel on these important outcomes.

The potential negative impact of any supplement given during the neonatal period on breastfeeding [10,18,19] necessitated close monitoring of feeding during the trial. In particular, the use of dextrose gel for treatment of hypoglycaemia has previously been reported in one small trial to reduce the volume of formula taken at the subsequent feed [20], although a larger, more recent trial was more reassuring and demonstrated reduced formula feeding rates at 2 wk after treatment dextrose gel [6]. We found no effects of prophylactic dextrose gel on measures of infant feeding (receipt of formula, delayed feeding, or breast feeding at discharge, on day 3, or at 6 wk). However, although all mothers of babies in our trial intended to breast feed, 55% of babies received formula before discharge, and 42% had not established full breast feeding by 72 h. This is perhaps not surprising given that 72% of mothers had diabetes and 48% underwent caesarean delivery; both are risk factors for delayed onset of lactation [18]. There are few comparative data. One study of women birthing in a university hospital in the US, the majority of whom intended to breastfeed and whose babies had no risk factors for hypoglycaemia, reported in-hospital formula supplementation in 47%, with the commonest indication being perceived insufficient milk supply [21]. Furthermore, delayed onset of lactation (≥72 h after birth) has been reported in 35% of healthy women [22].

We used a predefined assessment of limitations to assist with selecting the most appropriate dose, as we anticipated that more than one dose might be effective in preventing neonatal hypoglycaemia. As this prediction of similar efficacy proved correct, we aimed to select the dose that would be most acceptable to clinical staff and parents, with fewest potential adverse effects and best tolerated by the baby. Although the weightings of each component of the limitation score were assigned arbitrarily, they were based on our consensus estimate of clinical importance. This was helpful in clarifying that multiple doses were more likely than single doses to be associated with spilling, slower to administer, and considered messy by parents. However, there were no differences between dextrose and placebo gel groups.

The commonest risk factor for hypoglycaemia in participants in this trial was infant of a mother with diabetes. This was in large part because women pregnant with potentially eligible babies were approached antenatally, and we were able to identify women with diabetes more readily than those in other risk groups. Although this may be considered a potential weakness of this study, the incidence of neonatal hypoglycaemia was similar amongst the risk groups. Furthermore, prespecified subgroup analysis did not show any difference in efficacy of dextrose gel to prevent hypoglycaemia dependent upon the primary risk factor, although our trial was not powered to investigate this difference. Strengths of this trial are the low cost of the intervention and ease of administration of the gel, with potential for positive impact on neonatal health globally.

This trial was designed as a dose-finding trial, with the most effective dose in prevention of hypoglycaemia to be used to inform a subsequent multicentre trial to determine the effect on admission to NICU and on important long-term neurodevelopmental outcomes. Since efficacy of dextrose gel in prevention of hypoglycaemia was similar in all dosage groups, but limitations were more common in babies randomised to multiple doses, we have selected 0.5 ml/kg as the dose to be used in our ongoing trial of dextrose gel prophylaxis (Hypoglycaemia Prevention with Oral Dextrose [hPOD], ACTRN12614001263684) [23]. This also has the advantage of being the same as the dose shown to be effective and safe in treatment of neonatal hypoglycaemia [6], thus minimising any risk of confusion between prophylaxis and treatment in prescription and administration of gel in a clinical setting.

We have shown that in term and late preterm babies at risk of neonatal hypoglycaemia but without indication for NICU admission, the incidence of hypoglycaemia can be reduced by a single prophylactic buccal dose of 0.5 ml/kg 40% dextrose gel at 1 h of age, with an average of ten babies needing treatment to prevent one baby developing hypoglycaemia. It remains to be determined if this will result in other clinically important benefits in the short term and any effects on long-term health.

## Supporting Information

S1 Text Protocol. (PDF)

S2 Text CONSORT statement. (DOC)

## Abbreviations

CI: confidence interval
CONSORT: Consolidated Standards of Reporting Trials
hPOD: Hypoglycaemia Prevention with Oral Dextrose
IQR: interquartile range
NICU: neonatal intensive care unit
RR: relative risk
SD: standard deviation
