# Supplementary material for: Prophylactic Oral Dextrose Gel for Newborn Babies at Risk of Neonatal Hypoglycaemia: A Randomised Controlled Dose-Finding Trial (the Pre-hPOD Study)
Source: PLoS Med. 2016 Oct 25;13(10):e1002155. doi: 10.1371/journal.pmed.1002155 (PMC5079625; doi:10.1371/journal.pmed.1002155)
Supplement: S1 Text — (PDF) [file pmed.1002155.s001.pdf]

THE UNIVERSITY OF AUCKLAND
LIBRARIES AND LEARNING SERVICES

ResearchSpace@Auckland

Suggested Reference
Harding, J. E., Crowther, C. A., Alsweiler, J. M., Hegarty,J., Edlin, R., & Gamble, G. (2015). Study Protocol: Pre-hPOD – hypoglycaemia Prevention in newborns with Oral Dextrose. A randomised controlled dosage trial comparing different doses of prophylactic oral dextrose gel with placebo in newborn babies at risk of neonatal hypoglycaemia. (pp.18 pages).

Copyright
Items in ResearchSpace are protected by copyright, with all rights reserved, unless otherwise indicated. Previously published items are made available in accordance with the copyright policy of the publisher.

https://researchspace.auckland.ac.nz/docs/uoa-docs/rights.htm

Study Protocol

Pre-hPOD – hypoglycaemia Prevention in newborns with Oral Dextrose

A randomised controlled dosage trial comparing different doses of prophylactic oral dextrose gel with placebo in newborn babies at risk of neonatal hypoglycaemia.

hPOD STEERING GROUP:

Professor Jane Harding (Chair)

Professor Caroline Crowther

Dr Jane Alsweiler

Dr Joanne Hegarty

Dr Richard Edlin

Mr Greg Gamble (Study statistician)

COORDINATING CENTRE:

The Liggins Institute

The University of Auckland

Private Bag 92019

Victoria Street West

Auckland 1142

New Zealand

Telephone: +64 9 923 6691

Fax: +64 9 373 8763

Email: j.harding@auckland.ac.nz

Version record

Date	Version	Updates to this version
25/01/2013	1	Working copy for funding applications
18/03/2013	2	Addition of acronym ‘prehPOD’ for the initial dosage trial
24/06/2013	3	1. EPOC changed to iSTAT<br>2. CGM Gold changed to iPRO2<br>3. Carl Kushel named as Chair of SMC<br>4. Additional grant funding listed: HRC, AMRF, Lotteries and WMRF<br>5. ANZCTR registration number added
6/09/2013	4	Modified wording of DMC and SMC duties to ensure alignment with their Terms of Reference and addition of a further adverse event (systemic sepsis) as recommended by the DMC.
18/09/2014	5	Edited inclusion criteria to include 2.2kg (previously only >2.2kg):<br>2. Birth-weight ≥ 2.2 kg
23/12/2014	5.dosage	Edited to include only dosage trial (Pre-hPOD) protocol

Contents

Contents 3
Glossary 5
Title 6
  Pre-hPOD – hypoglycaemia Prevention in newborns with Oral Dextrose 6
Aims 6
  Research outcome 6
  Health impact 6
Study hypothesis: 6
Study design 6
Background 7
  Significance of the project 7
  Standard management 7
  Recent advances 7
  Selection of dose of prophylactic oral dextrose gel 7
  Continuous glucose monitoring 8
Research Plan 8
  Study Design 8
  Study Population 8
  Exclusion Criteria 8
  Primary Outcome 9
  Secondary Outcomes 9
  Informed Consent 9
  Discontinuation of randomised treatment 9
  Study interventions 9
    Dextrose Gel 9
    Surveillance 10
    Blood Glucose Analysis 10
    Continuous Glucose Monitoring (CGM) 10
  Adverse Events 10
  Follow-up after Primary Hospitalisation 11
Data Analysis 11
  Economic evaluation 12
  Power and Sample Size 12
Confidentiality & Data Security 13
Ethics 13
Trial Organisation 14

Steering Committee (SC) 14
Duties of the SC 14
Management Committee 14
Duties of the Management Committee 14
Independent Data Monitoring Committee (DMC) 14
Duties of the DMC 14
Study stopping rules 14
Safety Monitoring Committee (SMC) 15
Duties of the SMC 15
Resources 15
Trial Identification 15
References 16
Appendix I Limitations 18

Glossary

The following acronyms are used within the protocol.

Acronym	Full version
CGM	Continuous Glucose Monitoring
CGMS	Continuous Glucose Monitoring System
DRG	Diagnosis-Related Group
ICER	Incremental Cost-Effectiveness Ratio
LOS	Length Of Stay
NHI	National Health Index
NICU	Neonatal Intensive Care Unit[1]
NIH	National Institutes of Health
SCBU	Special Care Baby Unit

[1] This term is used throughout this protocol to refer to any unit where the baby is cared for away from the mother, and includes Special Care Baby Units (SCBU)

Title

Pre-hPOD – hypoglycaemia Prevention in newborns with Oral Dextrose

A randomised controlled dosage trial comparing different doses of prophylactic oral dextrose gel with placebo in newborn babies at risk of neonatal hypoglycaemia.

Aims

Research outcome

To determine the optimal dose of prophylactic oral dextrose gel to prevent neonatal hypoglycaemia when administered to newborn babies at risk.

Health impact

Approximately 30% of all New Zealand babies, or 21,000 babies a year, require multiple blood tests for neonatal hypoglycaemia under current guidelines.[1] Half of these babies (10,500 a year) will develop hypoglycaemia[1] and an unknown proportion of these will experience brain damage and developmental delay as a result. A much larger proportion will be given formula, potentially interfering with breast feeding and its associated benefits, including prevention of infectious, allergic, metabolic and developmental morbidity.

There are currently no strategies, beyond early feeding, aimed at preventing neonatal hypoglycaemia. This study will be the first to investigate whether neonatal hypoglycaemia can be prevented by a simple, cheap and painless intervention.

If successful, prophylactic dextrose gel could rapidly transform the management of neonatal hypoglycaemia, as there is considerable uncertainty both locally[2] and internationally[3,4] about how to best diagnose and manage this condition. However, prevention of hypoglycaemia by itself is only clinically useful if this in turn avoids further interventions for the baby such as admission to Newborn Intensive Care (NICU), and improves later outcomes. This dosage trial (pre-hPOD) is intended to determine an optimal dose of dextrose gel that will then be used in a multicentre randomised trial (hPOD) to determine whether prophylactic dextrose gel reduces the incidence of NICU admission.

Study hypothesis:

In newborn babies at risk of hypoglycaemia a 400mg/kg prophylactic dose of oral dextrose at one hour of age followed by 200mg/kg doses prior to the next three feeds, will be more effective than a 200mg/kg initial dose or pre-feed placebo doses in preventing hypoglycaemia.

Study design

Two centre randomised, double blind, placebo controlled dosage trial

Background

Significance of the project

Neonatal hypoglycaemia is common in the first few days after birth. Up to 15% of normal newborn babies will have low blood glucose concentrations[3], but approximately 50% of babies born at risk.[1] Despite this problem being recognised for more than 70 years, the best approach to diagnosis and management remains unclear.[4,7]

Glucose is the primary energy source for the brain, and neonatal hypoglycaemia is associated with brain damage and death.[6,8,9] Babies who are at risk for neonatal hypoglycaemia (infant of a diabetic, preterm, small or large for gestation) have an increased risk of developmental delay in later life. Our own preliminary data in 2-year-old Waikato children born at risk of hypoglycaemia shows that 38% have evidence of neurosensory impairment. Thus hypoglycaemia is a common, and the only readily preventable, cause of brain damage in the newborn.

Standard management

Blood glucose concentrations normally fall in the first 1-2 hours after birth, and then begin to rise again as babies mobilise their body stores of fat and glycogen and begin to feed. In some babies, this physiological fall in blood glucose concentration may persist and, if untreated, potentially may cause permanent brain damage. Since hypoglycaemia is often asymptomatic in babies, the recommended approach is to monitor blood glucose concentrations in all babies at risk, usually by repeated heel-prick blood samples, commonly 4 hourly, in the first 1-2 days.[14,15] This is painful for the baby and distressing for all concerned.

It is generally accepted that blood glucose concentrations < 2.6mmol/L require treatment.[6,16,17] Standard management of babies in whom low glucose concentrations are detected is to minimise the duration of hypoglycaemia and ensure the glucose is 'normalised' as quickly as possible.[9,18] This commonly requires admission to NICU for intravenous glucose, separating mother and baby and delaying the establishment of breast feeding as well as incurring high healthcare costs.

The American Academy of Pediatrics advises early identification of the at-risk baby and institution of *prophylactic* measures to prevent neonatal hypoglycaemia.[14] This is commonly achieved by early feeding, often with supplemental formula milk.[2,14] However, supplementing with formula milk has been shown to reduce longer term breastfeeding rates.[19] Furthermore, there are both human and experimental data indicating that supplementation of newborns in the first two weeks may have long-term effects on metabolic outcomes. Even brief periods of nutritional supplementation in preterm newborns result in altered control of blood pressure and insulin regulation in adolescence.[20,21] Thus, interventions that prevent hypoglycaemia without providing supplemental, artificial feeds may help maintain breastfeeding and also have benefits for both neurodevelopmental and metabolic outcomes.

Recent advances

We have demonstrated that *treatment* of neonatal hypoglycaemia with oral dextrose gel was more effective than feeding alone in reversing the hypoglycaemia, and also reduced the rate of NICU admission for this problem and reduced the rate of formula feeding at two weeks of age.[22] Importantly, the gel was well-tolerated, cheap, simple and safe to administer, and was acceptable to families and caregivers.

We therefore propose a randomised trial to determine if *prophylactic* oral dextrose gel given to newborns at risk can *prevent* neonatal hypoglycaemia and thus reduce NICU admission, improve breast feeding rates and reduce costs as well as potentially reducing the risk of later adverse outcomes.

Selection of dose of prophylactic oral dextrose gel

Currently, there are no data to inform the optimal dose of oral dextrose gel to prevent neonatal hypoglycaemia. Our previous study demonstrated that 200 mg/kg of oral glucose is effective in treatment of neonatal hypoglycaemia.[22] This is the same as the recommended dose of 2 ml/kg of 10% dextrose (200 mg/kg of glucose) given as an intravenous bolus to babies with hypoglycaemia unresponsive to supplementary feeds.[14] The normal glucose requirement of newborn babies is considered to be 4-6 mg/kg/min[21] but may be much higher in at-risk babies. Thus the same dose of 200 mg/kg of dextrose gel

given prophylactically might be expected to last for 40 minutes assuming a glucose utilisation rate of 5 mg/kg/min. For some babies this may be adequate, but for some at-risk babies the dose may need to be higher. We estimate that a 400 mg/kg prophylactic dose of oral dextrose may last for up to 50-100 minutes (assuming a glucose utilisation rate of 4-10 mg/kg/min), and that following this with 200 mg/kg doses before the next three feeds may potentially provide enough glucose to prevent hypoglycaemia during the transitional phase to normal physiological glucose production. However, these additional doses would mean additional interventions that may or may not be needed. Breast milk makes little contribution to total glucose requirements over this period, as volumes are initially small; as little as 13 ml/kg/day or 0.65 mg/kg/min glucose on day one and 40 ml/kg/day or 2 mg/kg/min of glucose on day two.[23]

Continuous glucose monitoring

By using continuous glucose monitoring during the dosage trial we will obtain a more detailed data on the effect of prophylactic dextrose on the physiological blood glucose nadir. Continuous glucose monitoring sensors have been shown to be well tolerated even in preterm infants.[24] We have previously shown that 81% of episodes of low interstitial blood glucose in neonates monitored with continuous glucose monitoring were not detected by interval blood glucose measurement.[25] A recent NIH workshop report recommended further studies 'to clarify the relationships among continuous glucose concentrations in the newborn, symptomatic hypoglycaemia, response to treatment, associated medical conditions, and longer term neurodevelopmental outcomes.[26]

Research Plan

Study Design

This will be a two-centre, randomised, placebo-controlled trial, comparing two doses of 40% dextrose gel with an identical appearing placebo gel, given either once only or an additional three times before feeds in the first 12 hours. The most effective, acceptable and safe dose of dextrose gel will be used in the subsequent multicentre trial (hPOD).

Study Population

Inclusion Criteria

Babies who are at risk of hypoglycaemia, defined as satisfying **at least ONE** of the following:

1. Infants of diabetic mothers (any type of diabetes)
2. Preterm (< 37 weeks' gestation)
3. Small (< 2.5 kg or < 10th centile on population or customised birthweight chart)
4. Large (> 4.5 kg or > 90th centile on population or customised birthweight chart)
5. Other risk e.g. maternal medication

AND satisfy **ALL** of the following:

1. ≥ 35 weeks' gestation
2. Birth-weight ≥ 2.2 kg
3. < 1 hour old
4. No apparent indication for NICU admission at time of randomisation
5. Unlikely to require admission to NICU for any other reasons e.g. respiratory distress
6. Mother intending to breast-feed

Exclusion Criteria

1. Major congenital abnormality
2. Previous formula feed or intravenous fluids
3. Previous diagnosis of hypoglycaemia
4. Admitted to NICU

5. Imminent admission to NICU.

Primary Outcome

Hypoglycaemia (any blood glucose concentration < 2.6 mmol/L in the first 48 hours)

Secondary Outcomes

1. Admission to NICU (NICU stay for >4 hours)
2. Admission to NICU for hypoglycaemia
3. Hyperglycaemia (blood glucose concentration of > 10 mmol/L)
4. Breastfeeding at discharge from hospital (full or exclusive)
5. Received any formula prior to discharge from hospital
6. Formula feeding at 6 weeks of age
7. Cost of care until discharge home
8. Maternal satisfaction (via telephone questionnaire at 6 weeks)

Informed Consent

Parents of babies who are likely to become eligible (maternal diabetes, likely late preterm birth, or anticipated high or low birth weight) will be identified through lead maternity carers and antenatal clinics and provided with an information sheet as early as is feasible. Written informed consent will normally be obtained before the birth by a member of the research team. Consent may be obtained immediately after birth if parents have been informed but not yet consented.

Eligible babies for whom consent has been obtained will be enrolled and randomised immediately after birth. Parents will be able to withdraw from the study at any time, with no effect on their baby's care.

Discontinuation of randomised treatment

The allocated treatment can be stopped at any time at the request of the parents, or by the neonatologist caring for the baby if (s)he feels that stopping the treatment would be in the best interest of the baby. The baby will still be followed up and analysed according to the intention-to-treat principle.

Study interventions

Dextrose Gel

Babies will be assigned randomly via an internet randomisation service to the dextrose or placebo group with priority stratification for collaborating centre and risk factor (i.e. maternal diabetes, preterm, small, large, and other). The staff member randomising the baby will receive a study number that will correspond to a pre-labelled study intervention pack which contains either 40% dextrose gel or identical appearing 2% hydroxymethylcellulose placebo gel. The randomisation process will also supply the volume of gel to be given from the pre-filled syringe. The inside of the baby's cheek will be dried with a gauze swab, and the study gel massaged into the buccal mucosa at one hour after birth, and if multiple doses, before the next 3 feeds, which would be 2-4 hourly according to standard hospital protocols.

The initial blood glucose concentration will be measured at 2 hours, as is current routine practice. Subsequent management will be according to hospital standard practices. If the baby becomes hypoglycaemic (blood glucose concentration < 2.6 mmol/L) then the hospital hypoglycaemia protocol will be followed, including the administration of supplementary feeds if relevant.

The study intervention drug (both dextrose gel and placebo) will be supplied by Biomed Ltd (Auckland, New Zealand). Each participating centre will have a supply of pre-labelled study intervention pack of gel held in a medications fridge.

Each baby will be randomly allocated to one of the dose arms as shown below.

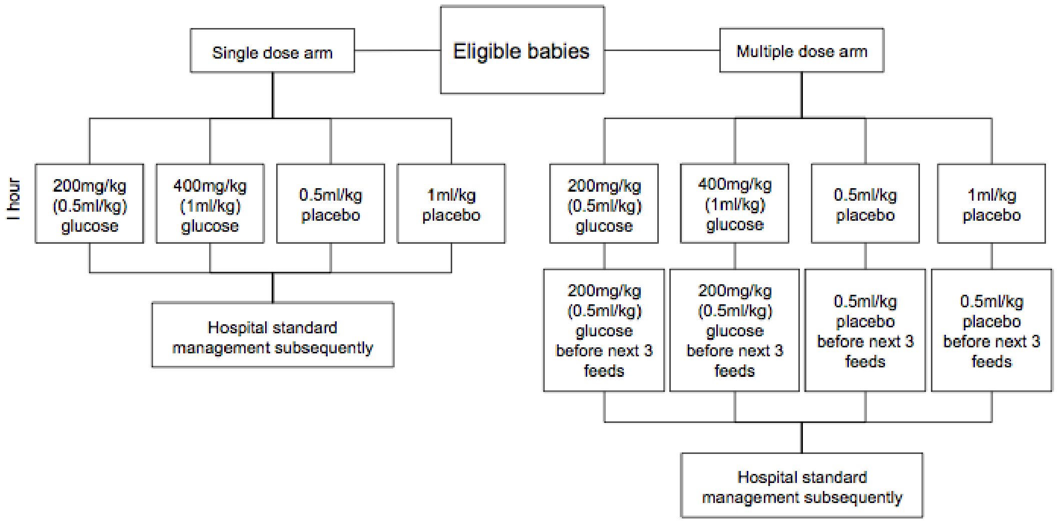

Figure 1. Dosage trial intervention

Surveillance

Babies will be monitored according to routine clinical practice. This includes pre-feed blood glucose measurements (taken before administration of the dextrose gel) 2-4 hourly for at least the first 12 hours, and until there have been 3 consecutive measurements > 2.6 mmol/L. All feeds taken by the baby will be recorded, including breastfeeds and any supplementary feeds. The parents will be telephoned at 6 weeks of age to determine feeding method and enquire about any adverse health events since discharge.

Blood Glucose Analysis

All blood glucose concentrations will be analysed by the gold standard glucose oxidase method, either with a portable blood glucose analyser (*iSTAT*, Abbott Laboratories, Abbott Park, IL USA) or a combined metabolite/blood gas analyser (ABL 700, Radiometer Ltd, Copenhagen, Denmark).

Continuous Glucose Monitoring (CGM)

All babies whose parent(s) give consent will have a continuous glucose monitor (CGM) sensor (*i*PRO™2, Medtronic, MiniMed, Northridge, CA,USA) inserted into the lateral aspect of the thigh as soon as possible after birth, to allow detection of the speed and duration of the effect of dextrose gel on glucose concentrations, and to allow more accurate detection of any episodes of hypoglycaemia. Data collected from the CGM monitors will be downloaded at the end of the study period, and are therefore not available to clinicians and cannot influence the clinical management of the babies. The sensors will remain *in situ* for 48 hours and will be calibrated at least twice a day.

Adverse Events

All adverse events will be reported to the Safety Monitoring Committee. Serious adverse events will be reported within 24 hours.

1. *Hyperglycaemia* (blood glucose concentration > 10 mmol/L). Dextrose gel has been very well tolerated in our previous study and in extensive clinical use for treatment of hypoglycaemia at Waikato Hospital, and we do not anticipate any problems. However, we will be using a larger dose of dextrose gel than used previously. All babies will be receiving routine blood glucose monitoring, and if hyperglycaemia occurs no further doses of gel will be administered.

2. *Late hypoglycaemia* (blood glucose concentration < 2.6 mmol/L for the first time after 12 hours of age). We will be giving the dextrose gel one hour after birth, when babies are recovering from the physiological nadir of blood glucose concentration that occurs after birth and is thought to be necessary to release hormones such as glucagon in order to stimulate glycogenolysis and gluconeogenesis and attain normal glucose homeostasis. Our continuous glucose monitoring data from our previous study suggest that this process will have been initiated by 1 hour of age, and that

giving dextrose gel at this stage should not interfere with the process. In our previous study, 20% of all hypoglycaemic episodes occurred beyond the first 24 hours.[1]

3. *Delayed feeding* (failure to establish breastfeeding without supplements by the end of day three): Repeated doses of dextrose gel have the potential to delay the establishment of regular feeding as the baby may not feel so hungry and therefore may not be motivated to feed. We will obtain this information by phone for those who have already gone home by this time.
4. *Systemic sepsis.*[27] Hyperglycaemia may increase the risk of infection.
5. *Seizures* (severe adverse event). Severe or prolonged hypoglycaemia can lead to neonatal seizures.
6. *Neonatal or infant death* (serious adverse event).

Follow-up after Primary Hospitalisation

Mothers of participants will be contacted by telephone on day three (if already discharged home) and 6 weeks after birth to complete a telephone questionnaire. This will include details of the current feeding regime, and at 6 weeks, maternal satisfaction with participation in the trial and health status of the baby. We will provide results when available to those who have informed us that they wish to be made aware of the outcome of the trial.

Data Analysis

The aim of this trial is to assist the hPOD Steering Committee to determine the optimal dose regimen for the main hPOD trial. This decision will be a synthesis of the dose required for adequate efficacy with a minimal burden of side-effects and pragmatically consider the ease of administration, simplicity and tolerance of the intervention and cost. Although sample size estimates were constructed on the basis of pairwise comparisons between placebo and incrementing doses this analysis will contribute to, but not exclusively frame the optimal dose recommendation.

Efficacy has been pre-specified as a reduction in the proportion of babies with at least one episode of hypoglycaemia from an estimated absolute 50% anticipated in the trial cohort to 25%. This dependent variable will be summarized as a percentage (± 95% confidence interval, binomial method) for each treatment arm.

To visualise the association between dose and outcome the cumulative administered dose for the single and multiple dose arms (ie 0, 200, 400, 800 and 1000 mg/kg) will be plotted as the independent variable. The response to single placebo dose (0 mg/kg) and multiple placebo dose (0 mg/kg x 4 feeds) for each dependent variable will be compared (Fisher's exact test) and the magnitude of any difference considered for clinical relevance. After consideration of the frequentist result and the potential clinical relevance of any potential difference in multiple and single placebo dose groups, either the placebo arms would be pooled into a single '0' mg/kg dose or if it is considered that multiple administrations of placebo adversely influences the dependent variables, the multiple dose placebo arm will be removed from the dose modelling analysis.

To incorporate comparison of each dose against placebo, logistic regression modelling the odds of hypoglycaemia for each cumulative dose of glucose and the multiple dose placebo arm relative to the single dose placebo arm will be plotted (with 95% confidence intervals) as the dependent variable against cumulative glucose dose (the independent variable). This analysis will adjust for risk factors for hypoglycaemia (ie sex, gestational age and mode of delivery). At the most rigorous analysis inspection of this function will determine that dose (or those doses) where the upper limit of the 95% confidence interval excludes a 50% reduction (the pre-specified efficacy endpoint).

It is likely that these data will yield a number of 'possible' doses where either the 95% confidence interval for the odds of reduced hypoglycaemia is significantly lower than the placebo dose (i.e. P odds of hypoglycaemia < 0.05) or that there is sufficient evidence that the odds of hypoglycaemia is likely to be at most 50% of the rate in the placebo arm, and consideration of these potential doses needs to be `traded off'. Therefore a complementary analysis of limitations at each dose level will be considered.

The odds of at least one limitation (tolerance or length of time to administer dose or messiness or hyperglycaemia or late hypoglycaemia or delayed feeding or unacceptability to parent(s)) for each cumulative dose arm relative to the placebo dose will be estimated (with 95% confidence intervals) and plotted and the likelihood that this estimate differs from the placebo arm reported. Additionally a limitation score, comprising the sum of weights assigned to the predetermined limitations (tolerance, time to administer dose, messiness, hyperglycaemia, late hypoglycaemia, delayed feeding and acceptability of trial intervention to parent, maximum score 18.5, see appendix I) will be summarised for each cumulative dose arm (median ± 95% confidence interval Mid-P method) and plotted. It is anticipated that about 5% of untreated babies might experience hyperglycaemia, 20% late hypoglycaemia and 25% delayed feeding. Seizures and infant death are not expected in this cohort.

To frame the discussion around the recommendation of study dose it is proposed that these data be examined four times: after 120, 240 and 360 patients have been accrued and at the end of recruitment. These milestones have been set after consideration of the likely width of the 95% confidence interval (ie precision) of estimates at each stage. In addition to providing data and safety monitoring information to the respective Data and Safety Monitoring Committees, it is proposed that these data be reported to those members of the hPOD Steering Committee **who have no contact with trial participants** so that adequate time is provided for the optimal dose recommendation/consideration to be made.

Additional data exploration will include plotting the mean (95% CI) time within the 48 hour follow up window where the continuous glucose monitor (CGM) glucose concentration falls below 2.6 mmol/L and the % of time where the CGM measured glucose concentration falls within the target range of 2.6-8.5 mmol/L. Both these dependent variables are currently under validation in other clinical trials.

Analyses will be performed using SAS (v9.3 SAS Institute Inc). All tests will be two tailed and $p < 0.05$ will be considered statistically significant. Since these are exploratory analyses no adjustment for multiplicity will be performed.

Economic evaluation

The cost-effectiveness of oral dextrose gel to prevent neonatal hypoglycaemia will be compared against usual care (no prophylaxis) within the period to discharge.

Intervention costs: A per-baby cost of dextrose gel will be based on the cost of the gel syringes, dispensing costs, and nurse time (in application, cleaning etc.). For the no prophylaxis option, a zero intervention cost will be assumed.

Other Hospital Costs: Resource utilisation will be obtained from a clinical record form identifying both length of stay (LOS) and relevant Diagnostic Related Group (DRG) code for the mother, plus any subsequent operative procedure (DRG), respiratory problem requiring treatment (DRG), and NICU/SCBU admission for the baby (plus LOS). Costs will be assessed using Ministry of Health cost weights and purchase unit prices. For the no prophylaxis option, the costs from the placebo gel arm will be used.

Cost-effectiveness will be assessed using incremental cost-effectiveness ratios (ICERs) formed in terms of an incremental cost per case of hypoglycaemia avoided. Uncertainty in these figures will be assessed using non-parametric bootstrapping (sampling with replacement) to form a distribution for the ICER, potentially including corrections for any differences in the composition of the trial arms in any confounding factors. This analysis will be presented using cost-effectiveness acceptability curves that identify the likelihood of each option being cost-effective for different values attached to reducing a case of hypoglycaemia.

Power and Sample Size

A trial of 415 babies (66 in each treatment arm, 33 in each placebo arm) and allowing for a 5% drop out rate, will have 80% power to detect a 25% relative reduction (absolute reduction of 50%) of neonatal hypoglycaemia from 50% to 25% (2-sided, alpha=0.05). 4 placebo groups are required to mask the difference in gel volume between the two doses of gel. However, the two single dosing placebo groups will be analysed together as one placebo group, as will the two multiple dosing placebo groups. Therefore, we will only require half the number of babies (33) in each of the 4 placebo arms.

Confidentiality & Data Security

Potential participants may be identified by screening by healthcare professionals involved in the care of the mother or baby antenatally. Any data collected antenatally (NHI number, expected date of delivery, entry criteria met) will be held by the primary investigator in a locked office or on a password protected computer.

Access to health information collected during the trial will be limited to recruitment staff (study nurses/midwives and site coordinators). A secure database will hold any identifiable data separately from trial data and access to this will be limited to the Investigators, Trial Coordinator and Data Manager. All trial data will be stored identified only by study number.

Research data and all study records will be retained for 10 years after the age of majority.

Ethics

Ethics approval has been obtained from the Health and Disability Ethics Committees of New Zealand.

Ethics reference 13/NTA/8

Trial Organisation

Steering Committee (SC)

Professor Jane Harding (Chair)

Professor Caroline Crowther

Dr Jane Alsweiler

Dr Joanne Hegarty

Dr Richard Edlin

Mr Greg Gamble

Duties of the SC

The Steering Committee will take overall responsibility for all aspects of the hPOD trial.

Management Committee

Professor Jane Harding (Chair)

Professor Caroline Crowther

Dr Jane Alsweiler

Dr Joanne Hegarty

Duties of the Management Committee

The Management Committee will be responsible for overseeing the day-to-day running of the trial

Independent Data Monitoring Committee (DMC)

Professor Frank Bloomfield (Chair)

Dr Katie Groom

Mr Greg Gamble (Trial statistician)

Duties of the DMC

1. To undertake interim analyses of the pre-hPOD (dosage) trial, including updated figures on recruitment, data quality, and primary outcome and safety data.
2. To make recommendations to the hPOD Steering Committee based on the above review or information from the Safety Monitoring Committee with regard to early cessation of the trial due to strong evidence of benefit or adverse effect (see discussion of stopping rules below).
3. Examine treatment effects in a blinded manner initially.
4. Advise the hPOD Steering Committee on any additional analyses to be undertaken to assess treatment effects during the trial.
5. Advise the hPOD Steering Committee on operational procedures affecting recruitment, treatment and follow-up.
6. The DMC can be contacted by collaborators and any others associated with the study if considered necessary. Requests for information from Third Parties will be discussed with the Steering Committee.

Study stopping rules

On evidence of interim data, the DMC may inform the Steering Committee that there is proof beyond reasonable doubt that:-

1. Prophylactic oral dextrose gel is either clearly indicated or contraindicated for all babies in the trial or for pre-specified subgroups of participants.

or

2. It is evident that no clear outcome will be obtained.

Safety Monitoring Committee (SMC)

Dr Carl Kuschel (Chair)

Dr Malcolm Battin

Dr Lindsay Mildenhall

Duties of the SMC

1. To commence review of all serious adverse events within 24 hours of receiving a report of this event.
2. To complete the review and report to the DMC and, if required, to the hPOD Steering Committee within 72 hours of receiving a report of a serious adverse event.
3. To determine for each serious adverse event reported whether the trial intervention was a causative factor in the event occurring.
4. To review each adverse event report and provide a written report to the DMC, and if required hPOD Steering Committee within 2 weeks of receiving the report.

Resources

Project funding has been obtained from Lotteries Health Board, Cure Kids, A+ trust, Auckland Medical and Research Foundation.

Trial Identification

The trial has been allocated Universal Trial Number (UTN) U1111-1138-0836 by the International Clinical Trials Registry Platform (ICTRP).

The trial is registered with the Australian New Zealand Clinical Trials Registry (ANZCTR) and allocated the ACTRN: ACTRN12613000322730. .

References

1.Harris DL, Weston PJ, Harding JE. Incidence of neonatal hypoglycemia in babies identified as at risk. J Pediatr 2012;161(5):787-91.

2.Harris DL, Weston PJ, Battin MR, Harding JE. A survey of the management of neonatal hypoglycaemia within the Australian and New Zealand Neonatal Network. J Paediatr Child Health 2009 Oct;50(10):E55-62.

3.Hay WWJ, Raju TN, Higgins RD, Kalhan SC, Devaskar SU. Knowledge gaps and research needs for understanding and treating neonatal hypoglycemia: workshop report from Eunice Kennedy Shriver National Institute of Child Health and Human Development. J Pediatr 2009;155(5):612-7.

4.Boluyt N, van Kempen A, Offringa M. Neurodevelopment after neonatal hypoglycemia: a systematic review and design of an optimal future study. Pediatrics 2006;117(6):2231-43.

5.Maayan-Metzger A, Lubin D, Kuint J. Hypoglycemia rates in the first days of life among term infants born to diabetic mothers. Neonatology 2009;96(2):80-5.

6.Lucas A, Morley R, Cole TJ. Adverse neurodevelopmental outcome of moderate neonatal hypoglycaemia. BMJ 1988;297(6659):1304-8.

7.Cornblath M, Hawdon JM, Williams AF, Aynsley-Green A, Ward-Platt MP, Schwartz R, et al. Controversies regarding definition of neonatal hypoglycemia: suggested operational thresholds. Pediatrics 2000 May;105(5):1141-5.

8.Burns CM, Rutherford MA, Boardman JP, Cowan FM. Patterns of cerebral injury and neurodevelopmental outcomes after symptomatic neonatal hypoglycemia. Pediatrics 2008;122(1):65-74.

9.Duvanel CB, Fawer CL, Cotting J, Hohlfeld P, Matthieu JM. Long-term effects of neonatal hypoglycemia on brain growth and psychomotor development in small-for-gestational-age preterm infants. J Pediatr 1999;134(4):492-8.

10.Silverman BL, Rizzo T, Green OC, Cho NH, Winter RJ, Ogata ES, et al. Long-term prospective evaluation of offspring of diabetic mothers. Diabetes 1991;40 Suppl 2:121-125.

11.Stenninger E, Flink R, Eriksson B, Sahlen C. Long-term neurological dysfunction and neonatal hypoglycaemia after diabetic pregnancy. Arch Dis Child Fetal Neonatal Ed 1998;79(3):F174-9.

12.Woythaler MA, McCormick MC, Smith VC. Late preterm infants have worse 24-month neurodevelopmental outcomes than term infants. Pediatrics 2011;127(3):e622-9.

13.Kerstjens JM, Bocca-Tjeertes IF, de Winter AF, Reijneveld SA, Bos AF. Neonatal morbidities and developmental delay in moderately preterm-born children. Pediatrics 2012;130(2):e265-72.

14.Adamkin DH. Postnatal glucose homeostasis in late-preterm and term infants. Pediatrics 2011;127(3):575-9.

15.Canadian Pediatric Society Fetal and Newborn Committee,. Screening guidelines for newborns at risk for low blood glucose. Paediatr Child Health 2004 Epub 2004/12/01;9(10):723-740.

16.Cornblath M, Ichord R. Hypoglycemia in the neonate. Semin Perinatol 2000 Apr;24(2):136-49.

17.Koh TH, Aynsley-Green A, Tarbit M, Eyre JA. Neural dysfunction during hypoglycaemia. Arch Dis Child 1988;63(11):1353-8.

18. Rozance PJ, Hay WWJ. Hypoglycemia in newborn infants: Features associated with adverse outcomes. Biol Neonate 2006;90(2):74-86.

19. Blomquist HK, Jonsbo F, Serenius F, Persson LA. Supplementary feeding in the maternity ward shortens the duration of breast feeding. Acta Paediatr 1994;83(11):1122-6.

20. Singhal A, Cole TJ, Lucas A. Early nutrition in preterm infants and later blood pressure: two cohorts after randomised trials. Lancet 2001;357(9254):413-9.

21. Singhal A, Farooqi IS, O'Rahilly S, Cole TJ, Fewtrell M, Lucas A. Early nutrition and leptin concentrations in later life. Am J Clin Nutr 2002;75(6):993-9.

22. Randomized trial of dextrose gel for treating neonatal hypoglycemia: the Sugar Babies Study. ; Abstract 3123.1; ; 2011.

23. Dollberg S, Lahav S, Mimouni FB. A comparison of intakes of breast-fed and bottle-fed infants during the first two days of life. J Am Coll Nutr 2001;20(3):209-11.

24. Beardsall K, Vanhaesebrouck S, Ogilvy-Stuart AL, Vanhole C, VanWeissenbruch M, Midgley P, et al. Validation of the continuous glucose monitoring sensor in preterm infants. Arch Dis Child Fetal Neonatal Ed 2013 Mar;98(2):F136-40.

25. Harris DL, Battin MR, Weston PJ, Harding JE. Continuous glucose monitoring in newborn babies at risk of hypoglycemia. J Pediatr 2010;157(2):198-202 e1.

26. Hay WWJ, Rozance PJ. Continuous glucose monitoring for diagnosis and treatment of neonatal hypoglycemia. J Pediatr 2010;157(2):180-2.

27. Australian and New Zealand Neonatal Network. ANZNN DATA DICTIONARY. Australia: National Perinatal Epidemiology and Statistics Unit (NPESU); 2015.

Appendix I Limitations

	Weightings
**Tolerated**	
Small spill of gel	0
Moderate spill of gel	1
Large spill of gel	2
**Time to administer dose** (estimated time in min to administer gel)	
<5 min	0
5-10 min	1
> 10 min	2
**Messiness**	
No	0
Yes	0.5
**Hyperglycaemia** (> 10 mmol/L)	6
**Late Hypoglycaemia** (< 2.6 mmol/L for the first time at > 12h of age)	6
**Delayed Feeding** (Failure to establish breast feeding without supplementation by the end of day 3)	2
**Acceptability of trial intervention to parent**	
Acceptable	0
Some inconvenience (e.g. delayed feeding when baby hungry)	0
Major inconvenience (e.g. baby very distressed by administration of gel)	1
Unacceptable	2
